# Early Onset Colorectal Cancer in Arabs, Are We Dealing with a Distinct Disease?

**DOI:** 10.3390/cancers15030889

**Published:** 2023-01-31

**Authors:** Adhari Al Zaabi, Asmaa Al Shehhi, Shaymaa Sayed, Humaid Al Adawi, Faris Al Faris, Omaima Al Alyani, Maitha Al Asmi, Abdulrahman Al-Mirza, Sathiya Panchatcharam, Maha Al-Shaibi

**Affiliations:** 1 College of Medicine and Health Sciences, Sultan Qaboos University, Muscat 123, Oman; 2 Pathology Department, Sultan Qaboos University Hospital, Muscat 123, Oman; 3 Surgery Department, Sultan Qaboos University, Muscat 123, Oman; 4 Ministry of Health, Muscat 393, Oman; 5 Ophthalmology Department, Oman Medical Specialty Board, Muscat 1422, Oman; 6 Research & Studies Section, Oman Medical Specialty Board, Muscat 1422, Oman; 7 Sultan Qaboos Comprehensive Cancer Care and Research Center, Muscat 123, Oman

**Keywords:** EOCRC, LOCRC, survival, MMR

## Abstract

**Simple Summary:**

The recent alarming increase in EOCRC incidence globally calls for research to understand the disease and its epidemiology from different geographical regions. Therefore, experts call for the inclusion of CRC patients from different geographical regions to understand the clinicopathological and molecular features of the disease. Our study is among the very few studies that evaluate the epidemiology and clinicopathological signatures and survival of EOCRC in the Arab population. Despite the reported significant difference in stage, molecular signature, and survival of EOCRC compared to LOCRC, our study showed no differences between the two groups. This could be explained either by a unique entity of the EOCRC in Arabs or the absence of a screening program for those above 50 years old.

**Abstract:**

Early-onset colorectal cancer (EOCRC) incidence is increasing worldwide. Efforts are directed to understand the biological and clinical signatures of EOCRC compared to late-onset colorectal cancer (LOCRC). EOCRC is thought to present differently across different ethnic groups and geographical regions. This study was an attempt to contribute with data from the Arab world toward the understanding of the clinicopathological parameters of EOCRC compared to LOCRC. Data from 254 CRC patients diagnosed at Sultan Qaboos University Hospital from the period 2015–2020 were studied. About 32.6% of all diagnosed CRC patients are below 50 years old, with no differences in gender distribution between EOCRC and LOCRC (*p*-value 0.417). Rectal involvement and tumor laterality were comparable among the two groups. Adenocarcinoma accounts for 83.3% and 94.2% of EOCRC and LOCRC, respectively. More mucinous and signet ring adenocarcinoma (8.3% each) were reported in EOCRC than LOCRC (2.9% and 2.2%, respectively). MLH1 and PMS2 loss are more common among LOCRC, but MSH6 loss is more frequent in EOCRC. The overall survival of EOCRC and LOCRC was comparable (median survival 64.88 and 67.24 months, respectively). This study showed comparable clinicopathological parameters between EOCRC and LOCRC from Arabs, which adds to the bigger picture of understand the disease.

## 1. Introduction

A plethora of published works have highlighted the alarming increasing trend of colorectal cancer among those younger than 50 years old, what is called Early-onset colorectal cancer (EOCRC) [1,2,3,4,5]. Colorectal cancer (CRC) is considered the third most common cancer and the leading cause of death worldwide in both genders [6]. Despite the slow and steadily decreasing in both the incidence and mortality of CRC in western countries, the incidence of early-onset CRC has nearly doubled since the 1990s [7]. According to the American Cancer Society, between 1994 and 2016, there was a noticeable decrease in the incidence of adult-onset CRC (>50 years) by 2% per year. At the same time, annual incidence rates of CRC have increased by more than 1.5% per year among patients 20 to 49 years old, and now early-onset CRC (EOCRC) comprises 10% to 18% of newly diagnosed CRC cases [8,9,10,11]. There is a prediction that by 2030 in the USA, 10% of all colon and 22% of all rectal cancers are expected to be diagnosed in patients <50 years old [12]. The overall reduction in CRC incidence has been mainly attributed to the higher uptake of screening tests that resulted in early detection and excision of premalignant lesions. The increase in risk factor awareness among the population has contributed as well [13]. On the other hand, the increase in EOCRC incidence has not yet been understood.

The status of CRC in Arab countries has not observed a similar global decline. In recent years, it has been observed that the incidence of colorectal cancer in the Arab population has been increasing. This could be largely attributed to improved cancer detection practices as well as the noticeable lifestyle changes and adoption of western dietary habits [14,15,16,17]. In Oman, CRC is considered the most common cancer in males and 2nd most common in females. The incidence has increased over the last 20 years [18], where almost 20% of all CRC patients are diagnosed below the age of 40 years [14].

Retrospective studies to understand EOCRC revealed that almost half of the cases are sporadic with no hereditary predisposition. Sporadic EOCRC has been found to present at most advanced stages and often in the distal colon and the rectum [19], and it exhibits mucinous and signet ring features or poorly differentiated histology [20]. Furthermore, they usually present with metastasis at diagn28osis and showed to have early disease recurrence and subsequently lower survival [21].

It is thought that sporadic EOCRC presents differently across different ethnic groups and geographical regions. Experts called for the inclusion of CRC patients from different geographical regions to understand the clinicopathological and molecular features of the disease [22].

Knowing that the majority of the information known about EOCRC comes from studies on the non-Arab population reflects the need for studies from different Arab countries to describe and report the unique characteristics of this disease entity. Understanding the status of EOCRC and its signatures would be of great significance for stakeholders to plan future cancer control plans tailored to this age group’s needs keeping in mind the increasing rates of EOCRC and the known Arab predominantly young population pyramid.

## 2. Materials and Methods

### 2.1. Material and Methods

Demographics and clinicopathological data, including the age of onset, gender, co-morbid, stage, grade, anatomical location of the tumor, rectal involvement, clinical TNM stage, histopathology data, i.e., tumor histological type and grade, pathological TNM stage, Mismatch Repair Protein (MMRP) status by immunohistochemistry including MLH-1, PMS-2, MSH-2, and MSH-6 were retrieved from the electronic health records of patients diagnosed with colorectal cancer at the Sultan Qaboos University Hospital, Oman from 2015 to 2020. The project was ethically approved by the ethical committee from the College of Medicine and Health Sciences.

### 2.2. Samples

All patients who were diagnosed with CRC or seen in SQUH in the period between 2015 and 2020 have been included. Patients who did not have follow-up records were excluded.

### 2.3. Definitions

Early onset CRC was defined as CRC diagnosed in patients <50 years old. Right colon tumors were defined as those arising in the cecum to the transverse colon, while left colon tumors were defined as those arising from the splenic flexure to the sigmoid and rectum. Rectal cancer is defined as those tumors located in the rectum up to 16 cm from the anal verge. Anorectal cancer was included since part of the rectum is involved. A tumor is considered to be Mismatch Repair Protein (MMRP) MMRP deficient (dMMR) if one or more of the MMRP proteins—MLH-1, PMS-2, MSH-2, and MSH-6—are not expressed. Otherwise, the status is regarded as mismatch repair proficient (pMMR) if there is intact nuclear expression of all four proteins.

## 3. Results

### 3.1. Demographics

There was a total of 254 colorectal cancer patients diagnosed between 2015 and 2020 at SQUH, with an average of 42 patients per year Table 1. Considering the number of EOCRC diagnosed per year, there is no significant difference across the years, with an average of 14 patients (32.6%) diagnosed yearly among all CRC patients. The ratio of young to old patients diagnosed per year is 1:2.

The average age was 40 years (range 19 to 49) year for the EOCRC and 63 years (range 50 to 86) for the LOCRC. In the EOCRC population, 50.6% were males, and 49.4% were females, whereas, among old patients, 56.3% were males and 43.7% were females (*p*-value 0.4) Table 2. There were no differences in gender distribution between the two age groups of CRC or rectal cancer (*p*-value 0.417 and 0.70, respectively). While young patients with rectal cancer had an almost equal gender distribution (58% young men vs. 42% young women), more men in the old group had rectal cancer (62% elderly men vs. 38% elderly women) (*p*-value < 0.065). There was no significant difference between EOCRC and LOCRC in terms of BMI or family history of colorectal cancer (*p*-value > 0.05). There was a significant difference in the rate of comorbid diseases such as diabetes mellitus, heart disease, and hypertension among LOCRC compared with EOCRC and the medication taken for such diseases (*p*-value < 0.05).

### 3.2. Disease Stage and the Anatomical Location at Diagnosis

The majority of EOCRC and LOCRC presented in stage IV, which is an advanced stage (48% and 42% respectively), followed by stage II (19%, 23%) and stage III (15%, 21%) Table 3. Only one young patient (age 47) was diagnosed with carcinoma in situ who did a colonoscopy test abroad per his request for abdominal bloating. There were six young patients and four old patients diagnosed with stage I disease. Considering rectal cancer, there were no differences in the proportions of stages between young and old patients (*p*-value = 0.08) Table 3.

### 3.3. Tumor Location and Histopathology

Table 4 shows the details of the tumor location and the histopathological findings of both EOCRC and LOCRC. In the EOCRC group, colon tumors were relatively more often located in the sigmoid colon (18, 23%) followed by rectosigmoid (12, 15.2%) and then transverse colon (8, 10.4%). There were no significant differences between rectal involvement among the EOCRC and the LOCRC (*p*-value 0.68). There is no difference in the tumor laterality between the two groups where the proportion of left-sided tumors is more than the right-sided tumors Table 4. When stratified by gender, old males tend to present with left-sided tumors compared to old females (*p*-value = 0.016). There is no gender difference seen in the sidedness of the tumor in the EOCRC group (*p*-value 0.369) Table 5.

As indicated in the table, the histologic subtype of EOCRC and LOCRC do not differ significantly (*p*-Value = 0.045). The most prevalent type in both categories was adenocarcinoma (NOS), accounting for 83.3% and 94.2% of EOCRC and LOCRC, respectively. EOCRC demonstrated a greater proportion of mucinous and signet ring adenocarcinoma (8.3% each) compared to LOCRC (2.9% and 2.2%, respectively).

Even though a higher pathological grade (G3) is more prevalent in EOCRC (10.2%) compared with 2.3% in LOCRC, this did not reach statistical significance (*p*-Value = 0.064). Both groups often have moderate tumor differentiation (G2).

Similarly, EOCRC and LOCRC do not differ much in terms of the pT stage, with the majority of cases presenting with pT3 followed by pT4.

Although EOCRC has a greater incidence of lymph node metastasis than LOCRC, the difference is not statistically significant (*p*-Value = 0.146).

There is no substantial difference between the two age groups concerning the prevalence of MMRP deficiency. In this study, we found that MLH1 and PMS2 loss is more common among older patients, but MSH6 loss is more frequent in younger patients.

### 3.4. Remission and Survival

**Figure 1:**
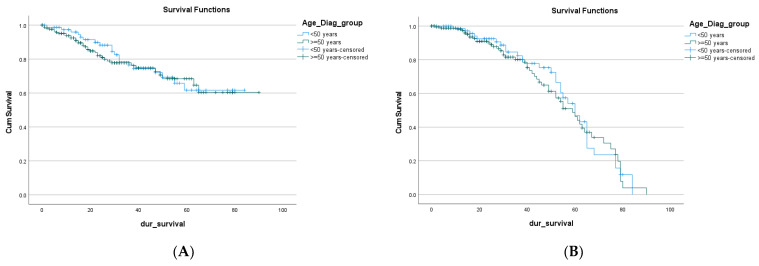
Mortality (**A**) and remission (**B**) of EOCRC and LOCRC.

There is no difference in the overall survival of EOCRC compared to the LOCRC (median survival 64.88 months and 67.24 months, respectively) 95% CI; *p* = 0.684 Table 6, Figure 1A.

The free overall survival was comparable between the two age groups with no significant difference (57.06 and 55.58 months, respectively, 95% CI; *p* = 0.653) Figure 1B.

### 3.5. Subcategories of EOCRC

Knowing that the literature is not consistent in defining the cut-off age for the EOCRC, different subcategories have been reported. Most of the published work defines it as CRC below the screening age, i.e., <50 years old, while others consider it as below 35 years old [19]. This unidentified age-group subdivision is considered one of the limitations for interpreting available published molecular and clinical data. Considering the reported findings that the major increase in the EOCRC in the USA was among those 20–35 years old who were found to have the worst prognosis [23], we have subdivided the EOCRC group into those <35 years old, and those 35–50 years old and run the comparison as shown in Table 7.

**Figure 2:**
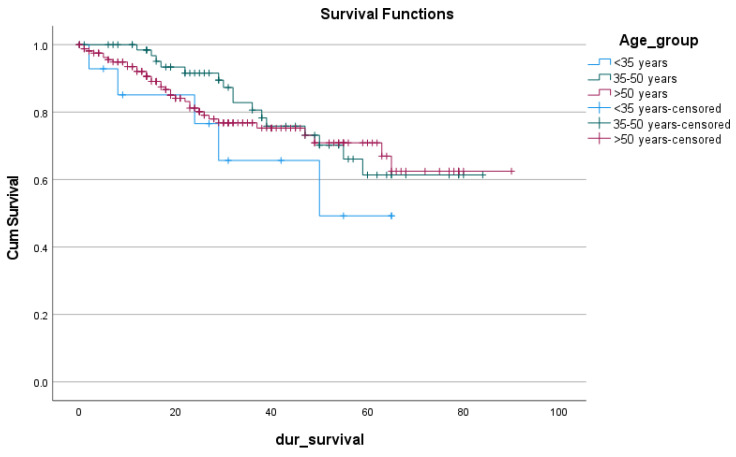
The survival rate of the CRC subgroups.

Table 7 showed no significant differences between the three subcategories in any of the clinicopathological determinants (*p*-value > 0.5). In Figure 2, the overall survival of those <35 is less than the other two subgroups (46.197, 66.059, 67.985, respectively, 95% CI; *p*-value 0.49) Table 8. This difference was not statistically significant considering the difference in the sample size of those <35 (14 patients) and those 35–50 years old (72 patients).

## 4. Discussion

It is not yet understood whether EOCRC represents a distinct disease entity from LOCRC. Efforts are directed toward deciphering the clinicopathological and molecular features of the disease across different geographical and ethnic areas. Experts are calling to include data from across the globe to understand the disease, which subsequently will improve its management. This study was an attempt to contribute with data from the Arab world toward the understanding of EOCRC, which is increasing at an alarming pace globally. Very few data are published on the molecular and clinical status of EOCRC from the middle east.

The present study showed that an average of 30% of all diagnosed CRC are younger than 50 years old, which is higher than what was reported earlier [3,14,24] but almost similar to some reports from the region [15,25,26]. There is no definite explanation for this high proportion of EOCRC in the region, but it is thought to be due to the general young population pyramid of these countries and the rapid adoption of a westernized lifestyle [23,24]. Smoking is becoming more common in the Gulf Cooperation Council countries, especially among the young [25,26], which could contribute to this high percentage of EOCRC. Data on the smoking and alcohol consumption of patients is incomplete, which prevented inferring an association. Another modifiable risk factor that could explain the high proportion of EOCRC in the region is the inactive lifestyle and the increase in the prevalence of obesity in the young population to an alarming level in the region [27]. The widespread use of prebiotics and probiotics in the region might explain the findings. Studies showed an increase in the consumption and availability of pro/prebiotics products in the Arabian peninsula in the past few years [20]. The epigenetic changes should never be underestimated in this content. Antibiotic overuse, climate change, environmental pollutants, and dysbiosis are all interrelated factors that need to be studied to better understand the disease and its increased incidence [28].

It is thought that COVID-19 might affect the cancer incidence and stage of the disease in the coming years due to the sudden disruption in cancer care services. Experts expect that such disturbances might lead to an upscale in cancer diagnosis and death post-COVID-19, called a “Tsunami of cancer”. There are also concerns that a “stage shift” will be seen in the post-COVID-19 era, where the delay in diagnosis and interrupted screening can lead to be patients diagnosed at advanced stages, which subsequently will impact survival negatively.

The government of the Sultanate of Oman took early and practical approaches and measures to tackle the pandemic and reduce its impact. For example, at Sultan Qaboos University Hospital, a tertiary center in Muscat, all non-urgent elective surgeries were temporarily suspended. Cancer-related diagnostic and surgical procedures were reduced from March to August 2020. Diagnostic gastroscopies and colonoscopies decreased by almost 80% during and after the first wave of COVID-19, and the numbers of colorectal surgeries were 50% lower than those recorded before March 2020. The data presented here did not show a significant difference in the number and stage of diagnosed CRC patients in 2020 compared to earlier years. The COVID-19 impact is thought to be more prominent a few years post-pandemic.

Prior studies have shown a significant association between EOCRC and metabolic diseases [29], which was not obvious in our study. Here metabolic diseases were significantly more common among LOCRC than EOCRC, which is mainly due to their direct link with age [26].

Previous studies showed that EOCRC significantly differs from LOCRC in being more left sided and with predominant rectal involvement [30], which was not prominent here since the proportions of right-sided and left-sided tumors were similar in both age groups with old males are more likely to present with left-sided tumors. The most prevalent anatomical location was rectosigmoid which is similar to previous reports [19,31]. There is no gender predominance in EOCRC compared to male predominance in LOCRC [31,32,33]. Here young males and females have an equal risk of getting diagnosed with CRC, but older males are significantly more prone to get the disease compared to old females. Males were also predominant in rectal cancer in the young age group. It is worth mentioning that rectal cancer constitutes up to 46% of all EOCRC cases in this study which is higher than reported earlier [4].

The majority of patients in both age groups presented with stage IV disease which is consistent with earlier data from Arab region [34]. This is most likely due to the absence of a national screening program in the country where the majority of patients are diagnosed after developing symptoms. It is even worse for younger patients because of the known delay in diagnosis that results from system, patient and physician related factors [35]. The majority of patients in both age groups presented with stage IV disease. This is most likely due to the absence of a national screening program in the country, where the majority of patients are diagnosed after developing symptoms. Prior studies have assumed that EOCRC presents in a more advanced stage due to its distinct aggressive molecular biology [5,8,36,37,38,39]. As a result, EOCRC patients are usually receiving aggressive treatments, which were not translated into an improvement in survival [5,11,40]. The survival of patients in this study did not show any differences, with a mean survival of 64.4 months and 67.2 for EOCRC and LOCRC, respectively. Interestingly those below 35 years of age are found to have a worse survival in this study. Considering prior reports from different geographical areas, the survival of this group of patients is significantly lower than their older counterparts [41,42,43] which might indicate a distinct biological signature that is worth studying. It is worth noting that only 14 patients in this study were in this age group which might not be reasonable to draw a statistical significance.

This study failed to find any statistically significant difference between EOCRC and LOCRC in terms of histopathological subtypes, tumor grade, pT stage, pN stage, as well as MMRP status.

The SEER analysis (1973–2005) found that 2.6% of colorectal carcinomas in young patients had signet ring histology [5], which is lower than the 8.3% of tumors with signet ring histology observed in our study. One reason for this difference is that we excluded neuroendocrine carcinoma cases which were included in SEER data. With regards to tumor differentiation, data from the SEER registry (1973–1999) demonstrated that colorectal carcinoma in young patients was more frequently higher grade compared to older patients [44]. We observed a similar finding, but it is not statistically significant. No difference was demonstrated between the two groups concerning the tumor stage pT or the number of lymph node metastases pN. This was similarly demonstrated in other studies [45]. Several reports showed that the proportion of MMRd tumors among early-onset CRC ranges from 19.7% to 41.0% 43, which is significantly higher than LOCRC [42,46]. However, in our current study, only 7.8% of EOCRC were classified as MMRd, with a similar proportion observed in LOCRC.

## 5. Limitation

Although our study is one of the few studies from the region that represents a five years description of EOCRC and LOCRC clinicopathological parameters, some limitations impact the comprehension of the study. First of all, this is a single institute study that prevents the generalization of the reported findings. Second, the amount of missing data, especially in the histopathology of the disease, affected the statistical analysis and the statistical conclusion from the presented data.

## 6. Conclusions

This study adds to the pre-existing literature to understand EOCRC. The most recent clinical and molecular studies revealed that EOCRC and LOCR are not biologically distinct diseases, but they do have different clinical presentations. Although few studies have shown that EOCRC has different clinical and molecular features when compared with LOCRC, this was not observed in our patient population, as there were no significant differences in the overall characteristics, tumor sidedness, or molecular profile of both EOCRC and LOCRC. This could be, in effect, a result of a smaller sample size and selection bias as we function as a referral center to specific parts of the country. Colorectal cancer cases are also managed in different centers in the country, and perhaps the pooling of all data will strengthen the results and help draw different results.

## Figures and Tables

**Table 1:** The proportion of EOCRC to LOCRC per year.

Year of Diagnosis	Number of Patients Diagnosed with CRC per Year	EOCRC < 50 Years	LOCRC ≥ 50 Years Old	Percentage of EOCRC Diagnosed per Year (%)
2015	39	14	25	36
2016	43	16	27	37
2017	45	13	32	29
2018	44	15	29	34
2019	41	11	30	27
2020	42	10	32	24
Total	254	79	175	31

**Table 2:** General demographics of CRC patients stratified by age.

Variables		Diagnosis Age		*p*-Value
		<50 Years *n* (%)	≥50 Years *n* (%)	
**Gender (CRC)**	Male	40 (50.6)	98 (56.3)	0.417
	Female	39 (49.4)	77 (43.7)	
**Gender (Rectal cancer)**	Male	21 (58)	46 (62)	0.065
	Female	15 (42)	28 (38)	
**Family History**	No	53 (67.9	119 (68.4)	0.707
	Yes	12 (15.4)	21 (12.1)	
	Unknown	13 (167)	34 (19.5)	
**BMI**	Missing	45 (57.0)	117 (67.2)	0.271
	<20	5 (6.3)	7 (4.0)	
	≥20	29 (36.7)	50 (28.7)	
**DM**	Absent	66 (83.5)	116 (66.7)	0.011
	Present	13 (16.5)	57 (32.2)	
	Not mentioned	-	2 (1.1)	
**HTN**	Absent	70 (88.6)	103 (59.2)	<0.001
	Present	8 (10.1)	68 (39.1)	
	Not mentioned	1 (1.3)	3 (1.7)	
**Heart disease**	Absent	76 (97.4)	142 (82.6)	0.001
	Present	2 (2.6)	27 (15.7)	
	Not mentioned	-	3 (1.7)	
**Medication**	**Metformin**			0.055
	Not given	69 (87.3)	132 (76.3)	
	Given	10 (12.7)	33 (19.1)	
	Not mentioned	-	8 (4.6)	
	**Aspirin**			<0.001
	Not given	76 (96.2)	145 (83.3)	
	Given	-	16 (9.2)	
	Not mentioned	3 (3.8)	13 (7.5)	

**Table 3:** Distribution of stage among (**A**) Colorectal cancer, (**B**) Rectal cancer.

**(A)** **Colorectal cancer**	**Stage**	**Overall Stage of CRC**		**Stage of EOCRC**		**Stage of LOCRC**		***p* Value**
		*n*	%	*n*	%	*n*	%	0.158
	**in situ**	1	0	1	1	0	0	
	**Stage 1**	10	4	6	8	4	2	
	**Stage 2**	55	22	15	19	40	23	
	**Stage 3**	49	19	12	15	37	21	
	**Stage 4**	112	44	38	48	74	42	
	**unknown**	27	11	7	9	20	11	
	**Total**	254	100	79	100	175	100	
**(B)** **Rectal cancer**	**Stage**	**Overall stage of rectal** **cancer**		**EOCRC**		**LOCRC**		***p* value**
		*n*	%	*n*	%	*n*	%	0.080
	**in situ**	0	0	0	0	0	0	
	**Stage 1**	5	5	4	11	1	1	
	**Stage 2**	17	15	5	14	12	16	
	**Stage 3**	24	22	5	14	19	26	
	**Stage 4**	52	47	20	56	32	43	
	**unknown**	12	11	2	6	10	14	
	**Total**	110	100	36	100	74	100	

Seven of the EOCRC patients (6%) and twenty of the LOCRC (14%) had unknown UICC stage, due to missing data with either unknown TNM stage.

**Table 4:** Clinicopathological comparison between early onset and late onset CRC.

Variables	Diagnosis Age		*p*-Value
	<50 Years	≥50 Years	
	*n* (%)	*n* (%)	
**Right**	19 (25%)	43 (25%)	1.00
**Left**	57 (75%)	130 (75%)	
**Rectum involvement**			
No	42 (53.8)	99 (57.2)	0.681
Yes	36 (46.2)	74 (42.8)	
**Mets by no. of involved organs**			
Non-Mets/missing	42 (53.2)	10 (58.0)	0.766
Single organ Mets	20 (25.3)	40 (23.0)	
Metastatic	17 (21.5)	33 (19.0)	
**Histology type**			
**Histology type (*n* = 209)**			
Adenocarcinoma (NOS)	60 (83.3)	129 (94.2)	0.045
Mucinous adenocarcinoma	6 (8.3)	4 (2.9)	
Signet-ring cell carcinoma	6 (8.3)	3 (2.2)	
Squamous cell carcinoma	-	1 (0.7)	
**Grade (*n* = 188)**			
1	4 (6.8)	6 (4.7)	0.064
2	49 (83.1)	120 (93.0)	
3	6 (10.2)	3 (2.3)	
**pT (*n* = 137)**			
IS	1 (2.1)	-	0.293
T1	2 (4.2)	-	
T2	3 (6.3)	9 (10.3)	
T3	30 (62.5)	59 (66.3)	
T4	3 (6.3)	6 (6.7)	
T4a	7 (14.6)	13 (14.6)	
T4b	2 (4.2)	2 (2.2)	
**pN (*n* = 123)**			
No	13 (34.2)	35 (41.2)	0.146
N1	3 (7.9)	19 (22.4)	
N2a	7 (18.4)	8 (9.4)	
N1a	2 (5.3)	4 (4.7)	
N1b	8 (21.1)	7 (8.2)	
N2	1 (2.6)	6 (7.1)	
N2b	3 (7.9)	3 (3.5)	
Nx	1 (2.6)	3 (3.5)	
**MLH1** **Immunohistochemistry** **(*n* = 182)**			
Intact	61 (95.3)	111 (94.1)	1.000
Lost	3 (4.7)	7 (5.9)	
**PMS2** **Immunohistochemistry** **(*n* = 182)**			1.000
Intact	61 (95.3)	111 (94.1)	
Lost	3 (4.7)	7 (5.9)	
**MSH2** **Immunohistochemistry**			
Intact	62 (96.8)	114 (98.3)	
Lost	1 (1.5)	2 (1.7)	
**MSH6** **Immunohistochemistry**			
Proficient	61 (96.8)	113 (97.4)	>0.05
Deficient	2 (3.2)	3 (2.5)	
**MMR (*n* = 182)**			
Deficient	5 (7.8)	9 (7.6)	1.000
Proficient	59 (92.2)	109 (92.4)	
**MSI (*n* = 21)**			
Positive	2 (16.7)	1 (11.1)	1.000
Negative	10 (83.3)	8 (88.9)	
**KRAS (*n* = 105)**			
Wild type	24 (58.5)	37 (57.8)	1.000
Mutant	17 (41.5)	27 (42.2)	
**BRAF (*n* = 60)**			
Wild type	17 (94.4)	40 (95.2)	0.824
Mutant	1 (5.9)	2 (4.8)	
**CEAugL_group (*n* = 224)**			
Normal (<2.5)	45 (60.8)	70 (46.4)	0.047
Abnormal	29 (39.2)	81 (53.6)	

**Table 5:** Gender distribution according to the sidedness of CRC between the EOCRC and LOCRC groups.

	EOCRC				*p*-Value	LOCRC				*p*-Value
	Right		Left			Right		Left		
	N	%	N	%		N	%	N	%	
Male	14	18	26	33	0.369	18	10	80	46	0.016
Female	10	13	29	37		25	15	52	30	
Total	24	30	55	70		43	25	132	75	

**Table 6:** Mortality and remission of CRC among the two age groups.

	Estimate (95% C.I.)	Standard Error	*p*-Value
**Mortality**			0.684
<50 years	64.88 (57.79–71.97)	3.62	
≥50 years	67.24 (60.86–73.63)	3.26	
**Remission**			0.653
<50 years	57.06 (51.34–62.78)	2.92	
≥50 years	55.58 (50.64–60.52)	2.52	

**Table 7:** Clinicopathological comparison between CRC subdivisions.

Variables	<35 Years	35–50 Years	>50 Years	*p*-Value
	*n* (%)	*n* (%)	*n* (%)	
**Stage**				0.252
Carcinoma in situ	-	1 (1.5)	-	
Stage 1	-	6 (9.2)	4 (2.7)	
Stage 2	4 (28.6)	12 (18.5)	39 (26.4)	
Stage 3	3 (21.4)	11 (16.9)	35 (23.6)	
Stage 4	7 (50.0)	35 (53.8)	70 (47.3)	
**Location**				0.17
Right	6 (46.2)	14 (20.3)	42 (25.1)	
Left	2 (15.4)	36 (52.2)	86 (51.5)	
Rectum	5 (38.5)	19 (27.5)	39 (22.8)	
Anal canal	-	-		
**Gender**				0.811
Male	7 (50.0)	37 (52.1)	94 (56.0)	
Female	7 (50.0)	34 (47.9)	74 (44.0)	
**Grade**				0.051
1	-	4 (7.4)	6 (4.9)	
2	8 (72.7)	47 (87.0)	114 (92.7)	
3	3 (27.3)	3 (5.6)	3 (2.4)	
**Status**				0.433
Alive	9 (64.3)	55 (77.5)	134 (79.8)	
Dead	5 (35.7)	16 (22.5)	34 (20.2)	

**Table 8:** Test of equality of survival distributions for the different age groups.

	Chi-Square	df	Sig.
Log Rank (Mantel-Cox)	1.411	2	0.494
Breslow (Generalized Wilcoxon)	3.192	2	0.203
Tarone-Ware	2.297	2	0.317

## Data Availability

The data presented in this study are available on request from the corresponding author.
